# Attitudes towards the Faecal Occult Blood Test (FOBT) versus the Faecal Immunochemical Test (FIT) for colorectal cancer screening: perceived ease of completion and disgust

**DOI:** 10.1186/s12885-016-2133-4

**Published:** 2016-02-13

**Authors:** Julie A. Chambers, Alana S. Callander, Rebecca Grangeret, Ronan E. O’Carroll

**Affiliations:** Psychology, School of Natural Sciences, University of Stirling, Stirling, Scotland FK9 4LA UK

**Keywords:** Faecal occult blood test (FOBT), Faecal immunochemical test (FIT), Colorectal cancer, Screening, Disgust

## Abstract

**Background:**

Colorectal cancer **s**creening is key to early detection and thus to early treatment, but uptake is often sub-optimal, particularly amongst lower income groups. It is proposed that the imminent introduction of the single-sample Faecal Immunochemical Test (FIT) in Scotland may lead to increased uptake as compared to the current Faecal Occult Blood Test (FOBT), but underlying reasons are yet to be determined. The aim was to evaluate attitudes and intentions towards completing the FIT compared to the current FOBT for colorectal cancer screening.

**Methods:**

A convenience sample of 200 adults (mean age 56.5, range 40–89; 59 % female) living in Scotland rated both the FOBT and the FIT with regard to ease of completion, perceived disgust and intention to complete and return (all measured on Likert-type 1–7 scale). Participants were randomised to be presented (via a face-to-face contact) with either the FIT or FOBT first.

**Results:**

Participants reported higher intention to complete and return the FIT versus the FOBT (mean difference 0.62, 95 % CI (0.44, 0.79)). Overall, 85.0 % (*n* = 170) of participants agreed or strongly agreed that they would intend to complete and return the FIT compared to 65.5 % (*n* = 131) for the FOBT (χ^2^ = 20.4, *p* < .001). The FIT was also perceived to be easier to complete (mean difference 0.85, 95 % CI (0.70, 1.01) and much less disgusting (mean difference 1.11, 95 % CI (0.94, 1.27)). Lower perceived disgust, higher socio-economic status and previous participation in any cancer screening were significant predictors of intention to complete the FOBT, whilst only higher perceived ease of completion predicted intention to complete the FIT.

**Conclusions:**

People reported higher intentions to complete and return a FIT than a FOBT test for colorectal cancer screening, largely due to a perception that it is easier and less disgusting to complete. The findings suggest that the introduction of the FIT as standard in the UK could result in a notable increase in screening uptake.

## Background

Around 1 in 20 people in the UK will develop colorectal cancer during their lifetime. Colorectal cancer is the third most common cancer in the UK, and is the second leading cause of UK cancer deaths, killing 16,000 people each year [1]. Screening aims to detect colorectal cancer at an early stage, in people with no symptoms, and can also detect non-cancerous polyps and adenomas which could develop into cancer over time, which can then be easily removed and reduce the risk of cancer developing. Thus, regular colorectal cancer screening can significantly reduce the risk of mortality.

In Scotland, all men and women aged 50 to 74 are offered screening every two years. At present, the Faecal Occult Blood Test (FOBT) is sent out by post for individuals to complete at home and post back to the National Bowel Screening Centre. The current FOBT kit involves collecting two pea-sized samples of faeces from each of three separate bowel movements within a period of 10 days and placing them under a flap (window) on a card. Once all 6 windows are completed, cards are posted back to the screening centre where they are tested for hidden traces of blood.

Of the 1.7 million people in Scotland that were invited for screening between November 2011 and October 2013, just over 960,000 (56.1 %) completed and returned their test [2]. In February 2012, the Scottish Government launched the Detect Cancer Early Programme (DCE) which aimed to increase early detection of the cancer by 25 % by the end of 2015. As part of this initiative, a new home screening kit (the Faecal Immunochemical Test or FIT), which involves taking and returning just one sample, will be introduced in Scotland over the next two years. As well as being apparently simpler to use, the FIT has greater diagnostic accuracy and has the potential to provide additional advantages in terms of a personalised risk (along with age/gender) of harbouring advanced neoplasia (colorectal cancer or high risk adenoma).

Between 2009 and 2011, the new FIT was piloted to around 66,000 people across two National Health Service (NHS) health boards in Scotland and return rates were compared to FOBT return rates in two different health boards [3]. The pilot study showed that FIT return rates were significantly higher within the two pilot health boards compared to the pre-FIT test period (i.e. 58-61 % versus 52-56 %) and significantly higher than FOBT return rates in the same period than the comparative health boards (i.e. 58-61 % versus 51-53 %). Although this suggests that rolling out the new test across Scotland may lead to an increase in screening uptake, no data was collected to determine the reasons for increased uptake.

One of the main differences between the current and the new screening test is that the new FIT involves taking just one sample on one occasion, compared to the FOBT which requires two samples to be taken on three separate occasions. Thus the process appears much simpler and it has been suggested that ease of completion plays a major role in the increased rate of return associated with the new FIT [4]. However, this study compared data from two separate periods when the FOBT (15 months) or FIT (9 months) was used as the standard kit during routine testing, and therefore did not provide a direct comparison between the two tests [4].

### Disgust

Liles et al. also reported that the increase in uptake for the FIT compared to FOBT was due to it being less unpleasant [4]. Disgust is a negative emotional reaction to unpleasant situations which appears to promote psychological and behavioural avoidance. Perceived or anticipated disgust has been determined as an important factor in colorectal cancer screening uptake [5, 6]. Individuals vary in their tendency to feel disgust with some experiencing it more often or more significantly than others. This suggests that individuals with greater *trait* disgust may be more impacted by *state* disgust than those who are less sensitive to disgust and therefore become more avoidant [7, 8]. In a study by Jones and colleagues [9], participants were asked to rate a list of barriers associated with different bowel cancer screening tests, including the FOBT. The top five barriers rated by participants in relation to the FOBT included the idea of 'not wanting to handle their own stool' and 'not wanting to keep stool samples on a card in the house' during the period of completing the test. Other studies have shown that reluctance to complete the FOBT is related to both disgust at the idea of handling stools, and concerns about posting samples in the mail [10, 11]. In comparison to the FOBT which involves putting stool samples on a piece of cardboard and retaining it in the house until all three samples have been taken, the FIT comes with a plastic test tube which conceals one sample that can be posted immediately after. This could lead to the new FIT being both easier to complete and perceived as less disgusting.

The present study provides a direct comparison of attitudes towards completing the FOBT and the FIT test with regard to ease of completion and perceived disgust. We used intention to complete and return the kit as a proxy measure of screening uptake. This was a within-subjects design controlled for presentation order. We hypothesised that participants would a) rate the FIT as easier to complete than the FOBT, b) rate the FIT as less disgusting to complete than the FOBT and c) report greater intentions to complete and return the FIT versus the FOBT.

## Methods

### Ethical approval

Ethical approval for the study was granted by the University of Stirling Psychology Ethics Committee, and all procedures were conducted in accordance with the Helsinki declaration (1975, revised 2000).

### Recruitment

Recruitment was via convenience sampling, including people known to the researchers and opportunistic recruitment from a local community health agency. Written informed consent was obtained from potential participants. All consenting participants completed all of the questionnaires.

### **Inclusion criteria**

Adults aged 40 years or more, living in Scotland, able to comprehend and complete self-report questionnaires written in English. We included adults aged under the age of 50 years (i.e. the age at which routine screening invitations are issued), as we wanted to explore the views of people who had never been invited to routine FOBT screening, and compare them to those of people who were likely to have received and/or completed a FOBT kit. There were no exclusion criteria.

### Design

A within-subjects randomised trial, with each participant rating both the FOBT and FIT screening kits. Order of presentation was randomised (prior to consent) via a computer generated random number table (https://www.randomizer.org/), to control for any priming effects on subject responses.

### Procedure

Data collection was carried out on a face-to-face basis with one of two researchers (AC and RG), either in the participant’s home or in a University room set aside for the research. Participants first completed a short demographics self-report questionnaire, which included age (also categorised into age band (1 = < 50 years old; 2 = > = 50 years), gender, postcode (to calculate Scottish Index of Multiple Deprivation (SIMD), which assesses deprivation based on geographic area via domains including income, employment, health, crime; we used SIMD quintiles where higher scores = lower deprivation, so 1 = most deprived and 5 = least deprived area), first-hand experience of major illness (Yes/No for: 1) self and 2) immediate family), whether they had previously participated in any cancer screening (Yes/No i.e. breast, cervical, colorectal, prostate), and whether they had previously completed an FOBT test.^1^ The latter was relevant for the over 50s group only, and thus was coded as ‘No’ for all under 50s. They were then presented with a blank version of one of the two screening kits (i.e. FOBT or FIT depending on randomisation) and the standard instructional information which would be posted out with the kit. Participants were requested to take as much time as they needed to read the instructions and familiarise themselves with the kit. They then completed a short questionnaire measuring attitudes (including ease of completion and test specific anticipated disgust items) towards completing the kit. The process was then repeated with the other test kit. Finally, participants completed a trait measure of disgust.

### Measures

#### Intention to complete and return, anticipated disgust, ease of completion

All items were scored on a seven-point Likert-type scale ranging from 1 (*strongly disagree*) to 7 (*strongly agree*). The 9-item questionnaire consisted of two items (Reliability found for FOBT: Cronbach’s α = 0.73; FIT: α = 0.59^1^) measuring intention to complete and return the kit (e.g. “If I was sent this test I would complete and return it”); three items (FOBT: α = 0.72; FIT: α = 0.54^2^) measuring ease of completion (e.g. “I would find it easy to complete this test”); and four items (FOBT: α = 0.80; FIT: α = 0.79) measuring perceived disgust (the ‘ICK-C’ e.g. “Completing this test would be an unpleasant task”). The measure was adapted from a questionnaire developed in a recent study [6].

#### Trait disgust

The Disgust Propensity and Sensitivity Scale – Revised (DPSS-R) [12] was used to assess two separate constructs that are thought to contribute to disgust reactions (i.e. trait disgust): disgust propensity (an individual's tendency to experience disgust) and disgust sensitivity (how unpleasant an individual considers experiencing disgust). Items were rated on a 5-point Likert-type scale (1 (*never*) to 5 (*always*)) (α = .80 for 6-item disgust propensity, α = .77 for 6-item disgust sensitivity). Mean scores were calculated for disgust propensity and sensitivity separately.

### Power analysis

It was estimated that 200 participants would be able to detect a small effect size in a repeated measures ANOVA (i.e. difference between FIT versus FOBT test: Cohen’s *f* = .13) with 80 % power (α = 0.05, two-tailed) [13].

### Statistical analyses

Data were coded and analysed using SPSS version 21, 2012. Primary analysis was a repeated measures ANOVA to test for differences in intention to complete and return, ease of completion and perceived disgust between the FOBT versus FIT screening kits, controlling for presentation order and age band (<50 years, > = 50 years). Correlation analyses were carried out to test for a relationship between the trait disgust measure and perceived disgust at completing the kit. Chi-squared tests were used to compare participants by categories of intention to complete the FOBT and/or the FIT kit. Two logistic regression analyses were carried out to test for factors predicting intention (agree/strongly agree vs other response) to complete and return: 1) the FOBT and 2) the FIT.

### Participants

There were 200 participants, 117 (58.5 %) female, 83 (41.5 %) males; mean age 56.5 years (*SD* = 11.28. range 40–89). Of the 200 participants, 99 were randomised to be shown the FOBT kit first and 101 to FIT kit first. There were no differences between randomised groups with regard to gender, SIMD quintile, previous or family illness, or screening history (Table 1). Participants shown the FOBT first were significantly older than those shown the FIT first (mean difference = 3.2, 95 % CI (0.1, 6.4)).

**Table 1:** Comparison of baseline variables by order of presentation of screening kit

	First presentation		
	FOBT	FIT	All
n	99	101	200
Age, mean (SD)	58.1 (11.4)	54.9 (11.0)	56.5 (11.2)
Gender: female, n (%)	60 (60.6)	57 (56.4)	117 (58.5)
^a^SIMD quintile: 1	15 (15.2)	18 (17.8)	33 (16.5)
2	17 (17.2)	15 (14.9)	32 (16.0)
3	23 (23.2)	14 (13.9)	37 (18.5)
4	12 (12.1)	22 (21.8)	34 (17.0)
5	32 (32.2)	32 (31.7)	64 (32.0)
Previous major illness:			
Yes	30 (30.3)	23 (22.8)	53 (26.5)
Family history of illness:			
Yes	47 (47.5)	48 (47.5)	95 (47.5)
^b^Previous cancer screening:			
Yes	78 (78.8)	73 (72.3)	151 (75.7)
Previous FOBT screening:			
Yes	40 (40.4)	44 (43.4)	84 (42.0)

Note: *FOBT* Faecal Occult Blood Test, *FIT* Faecal Immunochemical Test, *SIMD* Scottish Index of Multiple Deprivation; ^a^By definition we would expect around 20 % of the Scottish population to fall within each SIMD quintile; ^b^Previously screened for any cancer (breast, cervical, prostate, colorectal)

## Results

### Intention to complete and return, perceived disgust and ease of completion

We conducted a repeated measures ANOVA comparing intention, perceived ease of completion and disgust for the FOBT versus FIT kits (within-subjects) controlling for presentation order, age band and gender (between-subjects). There was an overall main within-subjects effect for type of kit (F(3, 190) = 62.8, *p* < .001, partial η^2^ = .50), but there were no between-subject effects for presentation order of kit (F(3, 190) = 0.8, *p* = .513, partial η^2^ = .01), age band (F(3, 190) = 0.9, *p* = .462, partial η^2^ = .01), or gender (F(3, 190) = 2.4, *p* = .069, partial η^2^ = .04). There were also individual effects for the FIT versus FOBT (See Table 2), with FIT having significantly higher intention and ease of completion scores and lower disgust than the FOBT. There were also individual significant effects of gender on ease of completion and disgust, with males perceiving the tests as easier to complete and less disgusting than females but there was no differences in intention (see Table 2). There were no other individual between-subject effects, nor any main or individual interaction effects between any of the variables.

**Table 2:** Estimated marginal means and individual effects of attitudes toward kit completion

	Mean (s.d)	95 % CI for mean	Mean (s.d)	95 % CI for mean	Individual main effects
*Type of kit*	*FOBT*		*FIT*		
Perceived ease of completion	5.32 (0.9)	(5.14, 5.49)	6.16 (0.5)	(6.06, 6.27)	F(1,192) = 60.7, *p* < .001, η^2^ = .34
Perceived disgust	3.91 (0.1)	(3.69, 4.12)	2.85 (0.1)	(2.65, 3.04)	F(1,192) = 95.5, *p* < .001, η^2^ = .43
Intention to complete kit	5.63 (0.1)	(5.42, 5.84)	6.24 (0.1)	(6.12, 6.36)	F(1,192) = 31.8, *p* < .001, η^2^ = .18
*Gender*	*Female*		*Male*		
Perceived ease of completion	5.61 (0.1)	(5.46, 5.76)	5.87 (0.1)	(5.68, 6.06)	F(1,192) = 4.6, *p* = .033, η^2^ = .023
Perceived disgust	3.57 (0.1)	(3.34, 3.79)	3.19 (0.1)	(2.89, 3.48)	F(1,192) = 4.2, *p* = .043, η^2^ = .021
Intention to complete kit	5.92 (0.1)	(5.74, 6.10)	5.95 (0.1)	(5.72, 6.18)	F(1,192) = 0.03, *p* = .990, η^2^ = .000

Note: Repeated measures ANOVA, adjusted for presentation order of kit, age band (<50 years, > = 50 years) and gender; *FOBT* Faecal Occult Blood Test, *FIT* Faecal Immunochemical Test;

### Trait disgust

The mean score on the DPSS-R propensity subscale was 2.65 (SD 0.6) and on the sensitivity subscale was 1.94 (SD 0.6). Both subscales were significantly correlated with perceived disgust at completing the FOBT (propensity r = .35, *p* < .001; sensitivity r = .23, *p* = .001) and the FIT (propensity r = .29, *p* < .001; sensitivity r = .19, *p* = .007).

### Logistic regression

Logistic regression was conducted separately for intention to complete the FOBT and the FIT test. Predictors included age, gender (female/male) and SIMD quintile (1–5, entered as categorical variable), perceived ease of completion, perceived disgust of completion, trait disgust propensity and sensitivity, previous major illness (yes/no), family illness (yes/no), previous participation in any cancer screening (yes/no), and having previously participated in FOBT screening (yes/no).

The unadjusted and adjusted (for all other variables in the model) odds ratios are shown in Table 3. In the unadjusted model for FOBT, SIMD (the two least deprived quintiles versus the most deprived), higher perceived ease of completion, lower disgust at completion, higher trait disgust propensity and sensitivity, having previously taken part in any cancer screening and having previously participated in FOBT screening were all significant predictors of intention to complete and return the kit. However, in the adjusted model only SIMD (least vs most deprived), lower perceived disgust, and having taken part in any previous cancer screening (Yes versus No) remained significant, with disgust being the strongest predictor. The adjusted model was significant (Cox & Snell R^2^ = 0.268, *p* < .001). For the FIT (see Table 3), significant unadjusted predictors were SIMD (the least versus the most deprived quintile), higher perceived ease of completion, lower disgust at completion, and higher trait disgust sensitivity (but not propensity). The adjusted model was significant (Cox & Snell R^2^ = 0.191, *p* < .001), with ease of completion being the only significant predictor of intention to complete the FIT (Table 3). The results of the logistic regression indicate that perceived ease of completion (FIT), perceived disgust at completing (FOBT) and any previous cancer screening participation (FOBT) appear to be more important predictors of intention to complete the kit than age, gender, SIMD (FIT only), experience of serious illness and trait disgust.

**Table 3:** Logistic regression of predictors of intention to complete and return the FOBT or FIT test, unadjusted and adjusted for other covariates in the model

	FOBT		FIT	
	Unadjusted Odds ratio	^a^Adjusted Odds ratio	Unadjusted Odds ratio	^a^Adjusted Odds ratio
Age	1.01 (0.99, 1.04	1.01 (0.97, 1.05)	1.01 (0.97, 1.04)	0.99 (0.94, 1.04)
GenderᅟᅟᅟᅟF	-	-	-	-
ᅟᅟᅟᅟᅟᅟᅟM	0.86 (0.48, 1.56)	0.96 (0.43, 2.14)	0.56 (0.24, 1.29)	2.18 (0.74, 6.42)
SIMD quintileᅟ1	-	-	-	-
ᅟᅟᅟᅟᅟ 2	1.06 (0.40, 2.81)	0.99 (0.31, 3.14)	2.63 (0.72, 9.61)	2.91 (0.60, 14.11)
ᅟᅟᅟᅟᅟ 3	2.50 (0.95, 6.60)	1.75 (0.55, 5.58)	3.09 (0.85, 11.24)	1.97 (0.42, 9.20)
ᅟᅟᅟᅟᅟ 4	2.88 (1.05, 7.88)*	2.77 (0.86, 8.95)	1.75 (0.54, 5.63)	1.51 (0.37, 6.24)
ᅟᅟᅟᅟᅟ 5	5.20 (2.05, 13.17)**	3.34 (1.16, 9.58)*	3.05 (1.02, 9.15)*	1.58 (0.42, 6.02)
Perceived ease of completion	2.15 (1.61, 2.88)***	1.37 (0.94, 2.01)	4.38 (2.45, 7.83)***	2.73 (1.33, 5.60)**
Perceived disgust	0.48 (0.37, 0.63)***	0.59 (0.41, 0.83)**	0.58 (0.43, 0.79)***	0.76 (0.50, 1.16)
Trait disgust propensity	0.52 (0.31, 0.88)*	1.08 (0.52, 2.23)	0.52 (0.27, 1.01)	1.18 (0.47, 2.97)
Trait disgust sensitivity	0.46 (0.28, 0.77)**	0.63 (0.32, 1.25)	0.37 (0.20, 0.69)**	0.52 (0.21, 1.30)
Experience of major illness (self)				
No	-	-	-	-
Yes	0.74 (0.39, 1.41)	0.68 (0.30, 1.55)	0.48 (0.21, 1.007)	0.40 (0.13, 1.21)
Family history of major illness				
No	-	-	-	-
Yes	0.82 (0.46, 1.47)	0.72 (0.36, 1.47)	0.65 (0.30, 1.42)	0.80 (0.31, 2.03)
^b^Previous cancer screening				
No	-	-	-	-
Yes	2.26 (1.17, 4.37)*	2.91 (1.06, 8.04)*	2.01 (0.88, 4.59)	3.52 (0.95, 12.99)
Previous FOBT screening				
No	-	-	-	-
Yes	2.12 (1.14, 3.92)*	0.96 (0.35, 2.01)	1.84 (0.80, 4.26)	1.13 (0.30, 4.23)

^a^Adjusted for all other covariates in the model; *p < .05, **p < .01, ***p < .001; ^b^Previously screened for any cancer (breast, cervical, prostate, colorectal), *SIMD* Scottish Index of Multiple Deprivation

## Discussion

A recent pilot of the Faecal Immunochemical Test (FIT) indicated that introduction of this as the first line screening test is likely to increase colorectal screening uptake compared to the guaiac Faecal Occult Blood Test (FOBT) which is currently used in the Scottish National Screening Programme [3]. The current study supported this in that participants reported much higher intentions to complete and return the new Faecal Immunochemical Test (FIT) versus the current guaiac Faecal Occult Blood Test (FOBT). It was hypothesised that the FIT would be perceived as less disgusting and easier to complete than the FOBT due to the new method and materials provided in the screening kit (i.e. test tube rather than cardboard sample card), and the elimination of the need to keep samples in the house over the testing period of up to ten days. In a direct comparison between tests, our findings confirmed that the FIT was perceived as being significantly easier and less disgusting to complete than the FOBT, adding to earlier findings by Liles et al. [4]. Importantly, this held both for adults who were not currently in the routine screening programme (under 50 years) and for those who were likely to have already received an invitation (50 years or greater).

The 4-item disgust scale (the ‘ICK-C’) had good internal reliability for both FIT and FOBT and was moderately correlated with trait disgust propensity and sensitivity, supporting its use as a measure of perceived disgust in colorectal cancer screening [6]. In the unadjusted logistic regression, perceived disgust and trait disgust sensitivity were significant predictors of intention to complete both the FOBT and the FIT, supporting the importance of disgust as a predictor of behavioural avoidance in contamination fear [14], and indicating that more disgust sensitive individuals may be disinclined to complete any test involving collection of faeces. When adjusting for all other variables (including trait disgust), perceived disgust at completing the kit, SIMD, and having attended any previous cancer screening were significant predictors of intention to complete and return the FOBT. In contrast, for the FIT, ease of completion was the only significant predictor, with neither perceived nor trait disgust being significant predictors of intention. This suggests that disgust may be a lesser barrier to uptake of the FIT and, providing it is viewed as easy to complete, factors associated with non-completion of the FOBT, including higher perceived disgust and lower socio-economic status may be less important in determining FIT uptake. The figure of 65.5 % who agreed or strongly agreed they would complete the FOBT is much higher than current actual rates of completion of the FOBT (i.e. 56.1 %), which is illustrative of the intention-behaviour gap, where changes in people’s intentions do not always translate into changes in actual behaviour [15]. It is, therefore, unlikely that the percentage actually completing the FIT would approach anything like the 85 % found to agree or strongly agree that they would complete and return the kit. Nonetheless, this represents a moderately large difference in intention to complete the FIT compared to the FOBT (i.e. 29.8 % more people), and even if, as suggested by Webb and Sheeran [15], it results in only a small to medium increase in behavioural change, it could have a major health impact at a national level. A 5 % increase in FOBT uptake is estimated to translate into approximately 11 additional cancers diagnosed per 100,000 of the target population [16]. Thus, the observed increase in intentions, coupled with the marked preferences for the FIT over FOBT, in terms of being less disgusting and easier to complete, suggests that the introduction of the FIT will translate into meaningful increases in screening uptake and resultant health benefits at a population level.

### Limitations

Limitations include the use of a convenience sample, which had a bias towards higher socioeconomic groups and female participants; and the fact that our outcome measures related to a hypothetical test (i.e. intention to complete and return and perceived disgust and ease of completion) and not actual return rate of completed kits. In addition, those randomised to view the FIT first were younger than the FOBT first group; however, neither age nor presentation order was associated with intention, ease or disgust, so this is unlikely to have affected our findings. We included participants aged 40–50 who would not have had a previous invitation to complete the FOBT as part of the Bowel Screening Programme. However, neither previous FOBT completion nor age predicted intention in the adjusted regression analysis for either test; in addition, the FIT was perceived as easier to complete and less disgusting than the FOBT by both under and over 50s. Thus, our findings apply to both those who may have received a previous FOBT kit as well as to those with no previous exposure.

## Conclusions

Our findings showed that almost 30 % more people said they would complete and return a FIT test compared to the current FOBT, which appeared to be due to it being perceived as easier and less disgusting to complete. Thus the present study indicates that the introduction of the FIT is likely to result in a notable increase colorectal cancer screening uptake.

## Abbreviations

DPSS-R: disgust propensity and sensitivity scale - revised
FIT: faecal immunochemical test
FOBT: faecal occult blood test
ICK-C: 4-item perceived disgust scale for colorectal cancer screening
NHS: National Health Service (UK)
SIMD: Scottish Index of Multiple Deprivation

## Footnotes

1 None of the sample had previously completed a FIT test.

2 Cronbach’s α was relatively low for the FIT intention and ease scales, which may be due to the limited number of items. Despite this it is still considered preferable to use multi-item measures for Likert-type scales, rather than single-item measures, which have very poor reliability [17]. We also conducted further examination of these scales which showed that removing the FIT ease item: ‘The instructions for this test are hard to follow’ actually increased Cronbach α to .58 for this scale. We repeated the analyses using the resulting 2-item scale for FIT ease, but all results remained significant as reported. Therefore for consistency of measures between the two tests, we report findings using the original 3-item FIT ease scale.
